# *Notes from the Field: Brucella abortus* RB51 Infections Associated with Consumption of Raw Milk from Pennsylvania — 2017 and 2018

**DOI:** 10.15585/mmwr.mm6915a4

**Published:** 2020-04-17

**Authors:** Joann F. Gruber, Alexandra Newman, Christina Egan, Colin Campbell, Kristin Garafalo, David R. Wolfgang, Andre Weltman, Kelly E. Kline, Sharon M. Watkins, Suelee Robbe-Austerman, Christine Quance, Tyler Thacker, Grishma Kharod, Maria E. Negron, Betsy Schroeder

**Affiliations:** ^1^Epidemic Intelligence Service, CDC; ^2^Pennsylvania Department of Health; ^3^New York State Department of Health; ^4^New Jersey Department of Health; ^5^Pennsylvania Department of Agriculture; ^6^Animal and Plant Health Inspection Services, U.S. Department of Agriculture, Washington, DC.

In December 2018, the Pennsylvania Department of Agriculture (PDA) and Pennsylvania Department of Health (PADOH) were notified of a New York patient with brucellosis caused by infection with *Brucella abortus* RB51, the live attenuated vaccine strain of *B. abortus* used to prevent brucellosis in cattle (*1*). Brucellosis is a serious zoonotic infection caused by the bacteria *Brucella* spp. The most common sign is fever, followed by osteoarticular symptoms, sweating, and constitutional symptoms (*2*). Without proper treatment, infection can become chronic and potentially life-threatening (*2*). The patient had consumed raw (unpasteurized) milk from dairy A in Pennsylvania.* In July 2017, Texas health officials documented the first human case of domestically acquired RB51 infection associated with raw milk consumption from a Texas dairy (*3*). In October 2017, a second RB51 case associated with raw milk consumption was documented in New Jersey^†^; the milk source was not identified at the time.

To determine the RB51 source for the New York case, PDA conducted an environmental investigation at dairy A in December 2018. PDA collected individual milk samples from all cows, excluding those known not to have been vaccinated against *B. abortus*, and from the bulk milk tank, which included milk pooled from all cows. All milk samples underwent polymerase chain reaction (PCR) testing and culture; whole-genome sequencing (WGS) was performed on patient and milk sample isolates. PDA conducted a traceback investigation of any cow with a milk sample that tested positive for RB51. PADOH worked with the raw milk cooperative that distributed dairy A’s milk to notify potentially exposed consumers and distributed notifications through Epi-X^§^ to identify cases.

Dairy A sold only raw milk and did not provide RB51 vaccination to cows born there (16 of the 30-cow herd). The remaining 14 cows were born outside the dairy and had inadequate vaccination records to determine whether they had received RB51. Because these cows might have been vaccinated, milk samples were collected from them. RB51 was detected by PCR and isolated in milk samples collected from the bulk tank and a single cow (cow 122). WGS identified two distinct RB51 strains shed by cow 122: one matched the 2018 New York patient’s isolate (3 single nucleotide polymorphisms [SNPs] different) and one, unexpectedly, matched the 2017 New Jersey patient’s isolate (1 SNP different). The two different RB51 strains were also shed from different quarters of cow 122’s udder.

Traceback revealed that cow 122 had received RB51 in 2011 and was purchased by dairy A in 2016. During 2016–2018, dairy A distributed raw milk potentially contaminated with RB51 to 19 states; PADOH notified those states’ public health veterinarians. PADOH provided a letter with RB51 information and brucellosis prophylaxis recommendations to the cooperative, which they distributed to dairy A customers. No additional cases were identified. Cow 122 was excluded from milk production, and serial PCR testing of bulk milk samples were subsequently negative for RB51.

Isolation of two different RB51 strains from different quarters of a cow’s udder has not previously been reported. These infections highlight the need to prevent RB51 infections. Raw milk consumption is also associated with serious illnesses caused by other pathogens, including *Campylobacter* spp., Shiga toxin–producing *Escherichia coli,* and *Salmonella* spp. (*4*). During 2007–2012, the number of raw milk outbreaks in the United States increased; 66 (81%) of 81 reported outbreaks occurred in states where raw milk sale is legal (*5*). Pregnant women, children, older adults, and persons with immunocompromising conditions are at greatest risk for infection.^¶^

To eliminate infection risk from milkborne pathogens, including RB51, all milk should be pasteurized. Because limited information is available about intermittent or continuous RB51 shedding among dairy cows, more research is needed to more fully understand this emerging public health threat for milk consumers. States can also consider the United States Animal Health Associations’ recommendations regarding the need for RB51 vaccination in areas where *B. abortus* is not endemic in wildlife.**

## Footnotes

* Retail sale of raw milk is legal in Pennsylvania. http://www.pacodeandbulletin.gov/Display/pacode?file=/secure/pacode/data/007/chapter59a/subchapFtoc.html&d=reduce.

† https://nj.gov/health/news/2017/approved/20171113c.shtml.

§ https://emergency.cdc.gov/epix/index.asp.

¶ https://www.cdc.gov/foodsafety/rawmilk/raw-milk-questions-and-answers.html.

** https://www.usaha.org/upload/Resolution/2018/Resolution_35_Use_of_RB51_Vacc.pdf.
